# Understanding the impact of 1q21.1 copy number variant

**DOI:** 10.1186/1750-1172-6-54

**Published:** 2011-08-08

**Authors:** Chansonette Harvard, Emma Strong, Eloi Mercier, Rita Colnaghi, Diana Alcantara, Eva Chow, Sally Martell, Christine Tyson, Monica Hrynchak, Barbara McGillivray, Sara Hamilton, Sandra Marles, Aziz Mhanni, Angelika J Dawson, Paul Pavlidis, Ying Qiao, Jeanette J Holden, Suzanne ME Lewis, Mark O'Driscoll, Evica Rajcan-Separovic

**Affiliations:** 1 Child and Family Research Institute, Molecular Cytogenetics and Array Laboratory, 950 West 28th Avenue, Vancouver, BC, Canada; 2 Department of Pathology, University of British Columbia, Vancouver, BC, Canada; 3 Department of Psychiatry, University of British Columbia, Vancouver, BC, Canada; 4 Human DNA Damage Response Disorders Group, Genome Damage & Stability Centre, University of Sussex, Brighton, UK; 5 Clinical Genetics Service, Centre for Addiction and Mental Health, Toronto, and Department of Psychiatry, University of Toronto, Canada; 6 Cytogenetics Laboratory, Royal Columbian Hospital, New Westminster, BC, Canada; 7 Department of Medical Genetics, University of British Columbia, Vancouver, BC, Canada; 8 Departments of Pediatrics and Child Health and Biochemistry and Medical Genetics, University of Manitoba, Winnipeg, MB, Canada; 9 Department of Physiology, Queen's University, Kingston, Ontario, Canada; 10 Department of Psychiatry, Queen's University, Kingston, Ontario, Canada; 11 Genetics and Genomics Research Laboratory, Ongwanada, Kingston, Ontario, Canada

## Abstract

**Background:**

1q21.1 Copy Number Variant (CNV) is associated with a highly variable phenotype ranging from congenital anomalies, learning deficits/intellectual disability (ID), to a normal phenotype. Hence, the clinical significance of this CNV can be difficult to evaluate. Here we described the consequences of the 1q21.1 CNV on genome-wide gene expression and function of selected candidate genes within 1q21.1 using cell lines from clinically well described subjects.

**Methods and Results:**

Eight subjects from 3 families were included in the study: six with a 1q21.1 deletion and two with a 1q21.1 duplication. High resolution Affymetrix 2.7M array was used to refine the 1q21.1 CNV breakpoints and exclude the presence of secondary CNVs of pathogenic relevance. Whole genome expression profiling, studied in lymphoblast cell lines (LBCs) from 5 subjects, showed enrichment of genes from 1q21.1 in the top 100 genes ranked based on correlation of expression with 1q21.1 copy number. The function of two top genes from 1q21.1, *CHD1L/ALC1 *and *PRKAB2*, was studied in detail in LBCs from a deletion and a duplication carrier. *CHD1L/ALC1 *is an enzyme with a role in chromatin modification and DNA damage response while *PRKAB2 *is a member of the AMP kinase complex, which senses and maintains systemic and cellular energy balance. The protein levels for *CHD1L/ALC1 *and *PRKAB2 *were changed in concordance with their copy number in both LBCs. A defect in chromatin remodeling was documented based on impaired decatenation (chromatid untangling) checkpoint (DCC) in both LBCs. This defect, reproduced by *CHD1L/ALC1 *siRNA, identifies a new role of *CHD1L/ALC1 *in DCC. Both LBCs also showed elevated levels of micronuclei following treatment with a Topoisomerase II inhibitor suggesting increased DNA breaks. AMP kinase function, specifically in the deletion containing LBCs, was attenuated.

**Conclusion:**

Our studies are unique as they show for the first time that the 1q21.1 CNV not only causes changes in the expression of its key integral genes, associated with changes at the protein level, but also results in changes in their known function, in the case of AMPK, and newly identified function such as DCC activation in the case of CHD1L/ALC1. Our results support the use of patient lymphoblasts for dissecting the functional sequelae of genes integral to CNVs in carrier cell lines, ultimately enhancing understanding of biological processes which may contribute to the clinical phenotype.

## Background

Copy number changes of 1q21.1 chromosomal region (OMIM 612474 and 612475) have been associated with variable phenotypes which include ID and/or autism [1,2], schizophrenia [3-5], congenital heart anomalies [2,6-8], dysmorphic features [1,6,7] or a normal phenotype [1,2]. Deletions and duplications of 1q21.1 were detected in 0.24% and 0.12% of cases respectively [9], and in 1/4737 controls [2]. The 1q21.1 critical region spans approximately 1.35 Mb (from 145 to 146.35 Mb, according to NCBI build 36) [2] and includes at least 12 genes. The cause of the phenotypic variability associated with 1q21.1 copy number variant (CNV) remains largely unexplained; however recent studies show that the presence of "two hit" CNVs can contribute to variability associated with CNVs that escape syndromic classification [10].

The impact of the 1q21.1 CNV, beyond the clinical description of affected subjects, is unknown. Traditionally, the functional impact of CNVs is studied in mouse models where expression changes in 83% of genes from CNVs were reported in at least one, but frequently in several, mouse tissues studied [11,12]. Mouse models of human microdeletion/microduplication disorders such as DiGeorge [13] and Smith Magenis syndrome [14], also helped to detect expression changes at the mRNA and protein levels of genes integral to CNVs and identify the critical candidate genes for the phenotype (e.g. transcription factors *Tbx1 *for DiGeorge and *RAI1 *for Smith Magenis syndrome). Subsequent studies of mutant forms of these genes in transfected human cell lines showed their abnormal function at the cellular level (i.e. changed transcriptional activity and/or abnormal sub-cellular localization/stability of the protein [15,16]). Unfortunately, functional consequences of genes integral to CNVs in cells/tissues from carriers are rarely studied, due to unavailability of appropriate human tissues and the rarity of patients with individual CNVs [17]. Nevertheless, in rare cases where human lymphoblasts were used to assess gene expression in CNV carriers, changes within the CNV and genome wide were noted [18,19] suggesting that peripheral blood cells can be used for assessment of the effect of gene copy number change. Subsequent studies of the function of genes showing expression changes in cells from CNV carriers have not yet been reported.

Our study aimed to understand the impact of the 1q21.1 CNV on gene expression genome wide as well as on the function of a selection of its integral genes in lymphoblasts cell lines from clinically well described subjects.

## Methods

### Subjects

Eight subjects were included in the study and their clinical description provided in Additional File 1, Table S1. They belong to three families (family A, B and C with 3, 3 and 2 subjects, respectively). Individuals A1, A2, A3, C1, and C2 were enrolled in a research based array CGH screening for pathogenic CNVs. The detailed criteria for enrollment were described in Qiao *et al*. (2010) [20]. The array CGH study was approved by the University of British Columbia Clinical Research Ethics Board. Subjects B1 and B2 were ascertained via a clinical genetics service. They had normal karyotypes and Fragile X testing. B1's brother, B3, was also ascertained through clinical genetic service because of the family history of 1q21.2 CNV.

### Whole Genome Arrays

The 1q21.1 CNV was detected in all subjects using initial lower resolution whole genome array analysis as previously described [20]. Seven of eight subjects were also analysed subsequently using the new Affymetrix Cytogenetics Whole-Genome 2.7 M Array (DNA was not available from B2 for high resolution array analysis). This higher resolution array contains approximately 400,000 SNP markers and 2.3 million non-polymorphic markers, with high density coverage across cytogenetically significant regions. Data was collected using either GeneChip^® ^Scanner 3000 7 G or GeneChip^® ^Scanner 3000 Dx and CEL files were analyzed using Affymetrix Chromosome Analysis Suite software (ChAS v.1.1). The annotation file used in our analysis can be found on the Affymetrix website, listed as ArrayNA30.2 (hg18). Additional CNVs detected with the high resolution array were compared with the Database of Genomic Variants http://projects.tcag.ca/variation for overlap with copy number variants in controls using previously described criteria for defining common variants [20].

### Fluorescent *in-situ *hybridization (FISH)

Rearrangements at 1q21.1 were confirmed by FISH following previously described protocols [21]. FISH probes used are listed in Additional File 1, Table S1.

### Whole genome expression

RNA from EBV (Epstein Barr Virus) transformed lymphoblastoid cell lines was used to study gene expression in subjects with a 1q21.1 microdeletion (A1-3), microduplication (C1 & C2), and in 3 normal controls. Transcript levels were assayed using a commercial whole genome expression array (Illumina, HumanRef-8 v3.0 Expression BeadChip) using standard protocols. Array hybridization, washing, blocking, and streptavadin-Cy3 staining were also done according to standard protocols (Illumina). The BeadChip was then scanned using an Illumina BeadArray Reader to quantitatively detect fluorescence emission by Cy3. Eight arrays were run in parallel on a single BeadChip. Each array contained ~ 24,500 well-annotated transcripts (NCBI RefSeq database Build 36.2, Release 22), present multiple times on a single array.

### Expression Data Analysis

Background-corrected intensity values were generated for each probe using GenomeStudio software (Illumina). Subsequent analyses were carried out in R http://www.R-project.org/. The data were quantile normalized and differential expression with respect to 1q21.1 copy number analyzed using limma [22], with Benjamini-Hochberg multiple test correction to control the false discovery rate (FDR). This yields a ranking of the genes used in subsequent analyses.

The ranking of genes from the 2.5 Mb and 5 Mb flanking regions of 1q21.1 (57 and 150 genes respectively) were examined in the full ranking provided by the analysis described above, and tested for enrichment using the Wilcoxon rank-sum test as well as the hypergeometric distribution considering just the 100 genes with the highest expression/1q21.1 copy number correlation.

### *In silico *functional analysis of top 100 genes

Genes which ranked highest (top 100 genes) in the expression/1q21.1 copy number correlation analysis were selected for further *in silico *functional analysis. An over-representation analysis (ORA) for Gene Ontology (GO) terms was performed using ermineJ http://www.chibi.ubc.ca/ermineJ/[23]. GO terms considered included biological processes, molecular functions, and cellular components. The ORA analysis was run using the following settings: gene set sizes were restricted from to 3-200 genes and best scoring replicates were used for any replicate genes in the datasets.

### Functional studies

#### Cell culture

EBV-transformed patient-derived LBCs were cultured in RPMI with 15% FCS (fetal calf serum), L-Gln and antibiotics (Pen-Strep) at 5% CO_2_. The Werner syndrome LBCs (WRN) were from a WRN syndrome patient homozygous for the p.Arg368X pathogenic mutation. A549 adenocarcinoma cells were maintained in MEM with 10% FCS.

#### Antibodies and Western blotting analysis

Anti-CHD1L (CHDL1 21703a), MCM2, phospho-S10-histone H3 and β-tubulin were from Santa Cruz. Antibodies against AMPKβ1, AMPKβ2 (4148), AMPKα and AMPKα-pT172, ACC, ACC-pS79 and RAPTOR-pS792 were obtained from Cell Signalling. Whole cell extracts were prepared by lysing cells in urea buffer (9 M urea, 50 mM Tris-HCl at pH 7.5 and 10 mM 2-mercaptoethanol), followed by 15 s sonication at 30% amplitude using a micro-tip (SIGMA-Aldrich). The supernatant was quantified by Bradford Assay. For CHD1L and AMPK-β2 expression, differing amounts of whole cell extracts were separated by SDS-PAGE and Western blotting signals were obtained following ECL (Pierce)-development. Densiometric quantification of scanned films was achieved using the Image J Software.

#### ATM- and ATR-dependent G2-M

G2-M cell cycle checkpoint analysis was carried out as previously described [17]. Briefly, following irradiation (3 Gy IR for ATM-dependent or 7 J/m^2 ^UV for ATR-dependent) cells were incubated for 4 h in the presence of 200 ng/mL of Demecolcine prior to swelling, fixation (Carnoy's) and staining as described below.

#### Decatenation Checkpoint Assay (DCC)

Exponentially growing LBCs were treated with 1 μM ICRF193 **(**Meso-4,4'-(3,2-butanediyl)-bis(2,6-piperazinedione) and 200 ng/mL of Demecolcine and incubated for 4 h. Cells were harvested, washed 1× in PBS and swollen in 75 mM KCl for 10 min before fixing with PBS containing 3% paraformaldehide, 2% Sucrose for 10 min. Following a PBS wash cells were cytospun on to polylysine coated slides and treated with 0.2% triton X-100 for 1 min before staining with an anti-phospho-histone H3 polyclonal antibody and secondary detection using Cy3-conjugated anti-rabbit. Nuclei were counterstained with 0.2 μg/mL 4,6-diamidino-2-phenylindole dilactate (DAPI) and viewed using Nikon E-400 microscope. Approximately 300 cells were counted per treatment.

#### CHD1L/ALC1 siRNA and ICRF193 treatment

*CHD1L/ALC1 *knock out in A549 epithelial lung cancer cells was done using 20 nM Darmacon SmartPool siRNA oligos with Metafectine as the transfection reagent according to the manufacturer's instructions. 20 h after addition of siRNA, cells were treated with 0.05 μM ICRF193 and 200 ng/mL of Demecolcine and incubated for 4 h. For chromosome spreads cells were swollen in 75 mM KCL (10 mins) and fixed in Carnoy's (methanol:glacial acetic acid 3:1) for 10 mins, washed (PBS), dropped onto slides and air dried prior to staining with Giemsa and imaged using a ZeissAxioplan microscope. Indirect immunofluoresence using anti-phospho-Ser10-Histone 10 was also carried out. At least 100 mitotic spreads were counted per treatment.

#### Pseudomitoses and Micronuclei determination

Cells with entangled chromosomes were considered to represent pseudomitoses. Their frequency was determined relative to interphase cells (mean no. of interphase cells counted per treatment = 300).

The levels of micronuclei (MN) were enumerated in cytochalasin B-induced binucleate [24] cells following exposure to and recovery from a low dose of ICRF193. The MN present in binucleate cells are derived from the previous cell cycle. Exponentially growing LBCs were treated for 16 hrs with 0.1 μM ICRF193. The drug was removed, cells washed in PBS and treated with cytochalasin B (1.5 μg/ml) for a further 24 hrs. Cells were pelleted, fixed in Carnoy's, stained with DAPI and, cytospun onto poly-L-Lysine coated slides and viewed using a Nikon E-400 microscope. At least 100 binucleate cells were enumerated per treatment.

## Results

### Clinical and genomic findings

**Figure 1:**
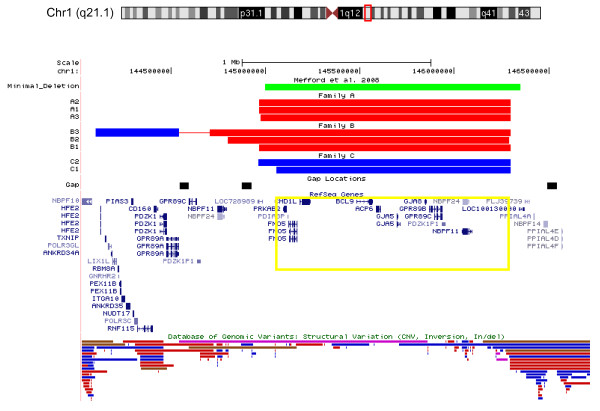
**Comparison of genomic overlap for 1q21.1 CNVs**. CNV breakpoints were determined using Affymetrix 2.7 M whole genome array for all subjects except B2 whose breakpoints were determined using a SignatureChip WG v1.1. Red bars indicate deletion of 1q21.1 region while blue bars indicate a duplication. The previously reported minimal deletion region is shown in green. Genes seen in the majority of our cases (core genes) are highlighted in yellow.

The clinical and genomic findings for all eight 1q21.1 CNV carriers are presented in Additional File 1, Table S1 and Figure 1. The clinical assessment included prenatal history and prenatal/newborn complications were documented in 5/8 cases. In addition, detailed clinical evaluation of 1q21.1 CNV carriers, both affected as well as those initially considered to be normal, was performed. This resulted in recognition of learning problems of various degrees in all studied subjects, although 2/6 subjects (A3 and C2) had very subtle learning difficulties as A3 did not complete secondary school training and C2 admitted having to work very hard to pass grades. Learning difficulties of variable degree were therefore common to all subjects, while other features varied, within and between families.

In family A, the phenotypes of three 1q21.1 deletion carriers showed different severity despite identical 1q21.1 gene content and almost identical 1q21.1 breakpoints (Additional File 1, Table S1 and Figure 1) as determined by high resolution 2.7 M Affymetrix array. In family B, phenotypes also differed between individuals, with individual B3 showing the least severe phenotype despite having the largest genomic imbalance which included both a deletion and a duplication. In family C, the affected proband (C1) inherited the duplication from his father, who was apparently normal but reported mild ADHD as child (not treated) and difficulties in passing grades in school.

The core genes seen in all subjects with a 1q21.1 CNV are *PRKAB2, PDIA3P, FMO5, CHD1L/ALC1, BCL9, ACP6, GJA5, GJA8, GPR89B, GPR89C, PDZK1P1*, and *NBPF11*. There were no secondary CNVs that could be considered pathogenic and contributing to the phenotype.

### Whole genome expression analysis

**Figure 2:**
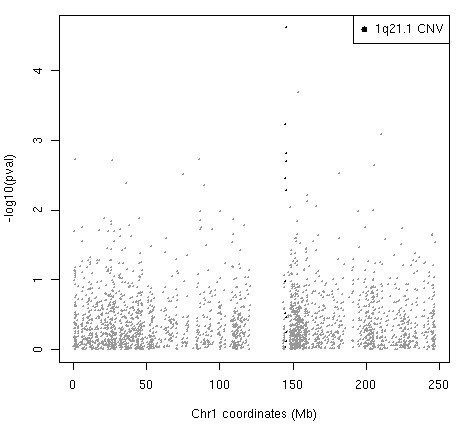
**Correlation of expression and copy number for probes from chromosome 1 expressed as log_10 _of the p values (see Methods)**. The probes from 1q21.1 region are in black.

Gene expression analysis was performed for 3 subjects with microdeletion (A1-3, from family A) two subjects with microduplication (C1 and C2 from family C) and 3 controls. Ranking of genes was based on correlation of expression changes and 1q21.1 copy number. Significant enrichment of gene transcripts from the 1q21.1 CNV (6/11 with probes on Illumina array) was detected within the top 100 genes in our 1q21.1 copy number/expression correlation analysis (Additional File 1, Table S2 and Figure 2). Transcripts from these genes (*PRKAB2, CHD1L/ALC1, BCL9, ACP6*, *GPR89A*, and *PDIA3P*) are ranked higher in our analysis than would be expected by chance (p = 2.5 × 10^-14^) and are positively correlated with 1q21.1 copy number with the exception of *PDIA3P*.

*CHD1L/ALC1*, a gene within the 1q21.1 CNV, was at the top of the correlation list, i.e. the correlation of its expression and copy number change was the least likely to have occurred by chance (p = 2.42 × 10^-5^, though not significant after multiple test correction). The p values for the correlation of expression and 1q21.1 copy number for all probes across all chromosomes is shown in Additional File 2, Figure S1. We did not find any evidence that the 1q21.1 CNV influenced expression of genes flanking the CNV (2.5 or 5 Mb windows; Wilcoxon rank-sum test and hypergeometric tests p > 0.2, see Methods). Gene Ontology enrichment analysis did not reveal any GO terms with more genes from the top 100 than would be expected by chance.

### Gene function analysis

**Figure 3:**
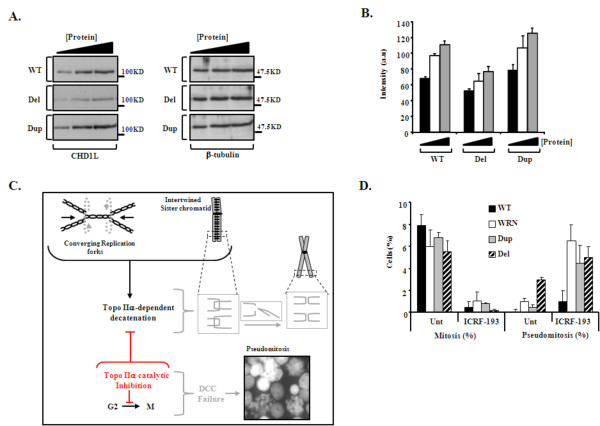
**Functional assays for CHD1L in patient cells. (A) **Left panels: Western analysis of CHD1L expression from wild-type (WT), 1q21.1 deletion (Del) and duplication (Dup) LBCs following titration of whole cell extracts. Right Panels: β-tubulin re-probe to confirm equal loading. **(B)**. Densiometric quantification of CHD1L expression from Western analysis from low (black bar), intermediate (white) to higher (grey) amounts of protein, from each line, using three separate determinations, normalized to β-tubulin loading (a.u. arbitrary units). p = 0.009 for Del and p < 0.005 for Dup compared to WT. **(C)**. The Decatenation Checkpoint (DCC). Unreplicated DNA sequences between converging replication forks undergo catenation and torsional tension which is normally relieved by Topoisomerase IIα (Topo IIα) which induces a transient DSB enabling decatenation (untangling). DCC activation in G2 prevents entry into mitosis until sister chromatids are fully separated. DCC can be activated by Topo II inhibitors arresting the cycle in G2 (indicated in red). DCC failure is monitored by the enumeration of '*pseudomitosis*' containing highly catenated (entangled) chromatids. Inset image shows typical pseudomitotic cells following treatment of the Del LBCs with the Topo II inhibitor, ICRF-193. **(D)**. Mitotic index (Mitosis %) and Pseudomitotic index (Pseudomitosis %) for untreated (Unt) LBCs or ICRF-193 treated, for wild-type (WT), Werner's syndrome (WRN), Dup and Del containing LBCs. WRN LBCs are known to be defective in DCC activation. Data presented indicates the mean ± s.d of three separate determinations. p < 0.005 for reduction in Mitosis (%) Unt compared to ICRF-193 and p < 0.005 for increase in Pseudomitosis (%) Unt compared to ICRF-193.

Gene function analysis was performed using LBCs from B1 and C1. B1 represented the 1q21.1 deletion (Del) and C1 represented the 1q21.1 duplication (Dup). Two genes, *CHD1L/ALC1 *and *PRKAB2*, from 1q21.1 were studied because they ranked highest in the expression/1q21.1 copy number correlation analysis (*CHD1L/ALC1 *position 1 and *PRKAB2 *position 10) and because they have functions in relevant cellular processes (see below and discussion for details). The protein expression of these genes was determined using Western blotting in patient LBCs. Reduction of protein level for both *CHD1L/ALC1 *and *PRKAB2 *was seen in the LBCs with 1q21.1 Del and an increase in the LBCs with 1q21.1 Dup in comparison to the control (Figure 3A and 3B and 5A respectively).

#### Functional assays for CHD1L/ALC1

*CHD1L/ALC1 *(hereafter referred to as *CHD1L*) has been implicated in chromatin remodeling and DNA relaxation process required for DNA replication, repair and transcription [25]. Both depletion and over-expression of CHD1L have been implicated in impaired chromatin remodeling during DNA single strand break repair [26] suggesting that it is a dosage-sensitive gene with a role in DNA Damage Response (DDR). The DDR incorporates DNA repair processes as well as signal transduction processes and is coordinated by two protein kinases ATM (Ataxia Telangiectasia Mutated) and ATR (Ataxia Telangiectasia Mutated Rad3-related) that sense DNA damage and co-ordinate appropriate cell cycle checkpoint activation, DNA repair and apoptosis [27].

We set out to probe DDR function in 1q21.1 CNV LBCs by initially examining the ATM and ATR-dependent G2-M checkpoint via mitotic index enumeration following ionising radiation (IR; for ATM-dependent arrest) or UV irradiation (for ATR-dependent arrest) respectively. LBCs with a deletion or duplication of 1q21.1 exhibited normal arrest, as evidenced by an increase in G2 cells and decrease in mitotic cell index, suggesting functional ATM and ATR-dependent checkpoint activation (data not shown). But, in the course of this analysis we noticed elevated levels of '*pseudomitosis' *in LBCs containing 1q21.1 Del and Dup containing cell lines, which prompted more detailed analysis of their frequencies in the 1q21.1 Del and Dup cell lines. Pseudomitotic cells exhibit catenated entangled chromatids and their presence indicates sub-optimal Decatenation Checkpoint (DCC) activation (Figure 3C). The DCC is a functional cell cycle checkpoint, involving proteins such as ATR, ATM, WRN, MDC1, BRCA1 and RAD9, that delays cells in G2 phase until DNA is fully decatenated [28]. Chromosome catenation is a normal by-product of DNA replication as replication forks attempt to merge producing intertwined catenated sister chromatids (Figure 3C). Topoisomerase II alpha (Topo IIα) specifically functions to decatenate/untangle these chromosomes by transient introduction of a DNA double strand break (DSB) allowing one strand to pass through the other thereby facilitating completion of DNA replication and faithful chromosome segregation (Figure 3C). DCC can be activated following treatment with a bisdioxopiperazine Topo II catalytic inhibitor that prevents Topo-II-dependent DSB formation (e.g. ICRF193).

**Figure 4:**
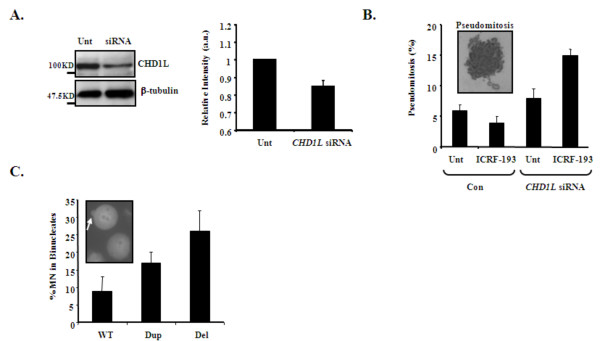
**Consequences of limiting CHD1L levels by siRNA. (A).** Careful titration of *CHD1L* siRNA conditions were undertaken in A549 so as to mimic haploinsufficient expression of CHD1L. Left panels:These show the Western analysis of whole cell extracts from Untreated (Unt; mock-treated) control whereas siRNA indicates cells treated with *CHD1L *siRNA. β-tubulin expression was monitored to confirm equal loading. **Right graph: **Densiometric quantification of CHD1L expression, normalized to β-tubulin loading from three separate siRNA experiments. The degree of CHD1L reduction is very similar to that observed from the 1q21.1 deletion (Del) containing LBC (Fig 3A and B). Data represents the mean ± s.d. of three separate experiments (a.u. arbitrary units). **(B)**. Inset image shows a typical catenated pseudomitotic cell following *CHD1L *siRNA-mediated knockdown in A549 treated with Topo II inhibitor (ICRF-193). The % pseudomitosis were enumerated under various conditions in A549 following *CHD1L *siRNA-mediated knockdown to mimic haploinsufficiency. Unt (untreated; not treated with ICRF-193), ICRF-193 (treated with ICRF-193), Con (control siRNA scrambled oligo), *CHD1L *siRNA (treated with siRNA to mimic *CHD1L *haploinsufficiency). Data represents the mean ± s.d. of three separate experiments and p < 0.005 for increase in Pseudomitosis (%) following *CHD1L *siRNA. **(C)**. Inset image shows micronuclei (MN). The % of ICRF-193-induced MN in binucleate cells were determined in wild type (WT), Dup and Del containing LBLs following a 24 hr recovery from an overnight treatment with ICRF-193. Data represents the mean ± s.d. of three separate experiments and p = 0.02 for increase % MN in binucleates for Dup and p < 0.005 for Del containing LBCs.

Interestingly, we found that LBCs carrying a Del or Dup of 1q21.1 failed to arrest in G2 following Topo II inhibition, and instead, exhibited elevated pseudomitosis similar to *WRN-*defective cells from a patient with Werner syndrome (OMIM #277700, Figure 3D), which are known to exhibit defective DCC activation [29]. Considering that CHD1L functions as a chromatin remodeler [26], and that catenated chromosomes are a conceivable outcome of an inability to efficiently manipulate chromatin structure, we sought to determine whether reduction of CHD1L specifically could underlie this phenotype. Using careful titration of CHD1L siRNA in A549 cells so as to mimic the patient LBC situation (Figure 4A), we found that modestly reduced CHD1L was indeed associated with impaired DCC activation following Topo II inhibition and resulted in increase in number of pseudomitoses (Figure 4B). These data describe a novel consequence of limiting CHD1L levels.

Failure of the DCC can also ultimately result in chromosome breakage and elevated levels of genomic instability as evidenced by increase in micronuclei [30,31]. Consistent with DDC failure observed in 1q21.1 Del and Dup containing LBCs, we found elevated levels of micronuclei in both LBCs following prolonged treatment (16 hrs) with ICRF193, although to a greater extent in the 1q21.1 Del containing LBCs compared to the 1q21.1 Dup containing LBCs (Figure 4C). Nevertheless, these data are consistent with a failure to efficiently activate the DCC and with elevated levels of DSBs which manifest as micronuclei in these cultures (Figure 4C). There was no evidence of spontaneous chromosome instability or increased micronuclei formation in the 1q21.1 Del and 1q21.1 Dup containing cell lines based on analysis of solid stained and G banded patient chromosomes and nuclei after short term culture.

#### Functional assays for PRKAB2

AMP-activated protein kinase (AMPK) senses and regulates systemic and cellular energy balance by regulating food intake, body weight, and glucose and lipid homeostasis [32]. It also plays an important role in negatively regulating the mTOR pathway that functions to control ribosome and protein biosynthesis [33]. AMPK is a heterotrimeric complex composed of a catalytic α-subunit, a regulatory β-subunit and an ADP/ATP-binding γ-subunit [34]. Furthermore, several isoforms of each subunit exist (α1, α2, β1, β2, γ1, γ2, γ3) thereby enabling the generation of multiple distinct heterotrimeric complexes. *PRKAB2 *encodes the β2-isoform of AMPK.

**Figure 5:**
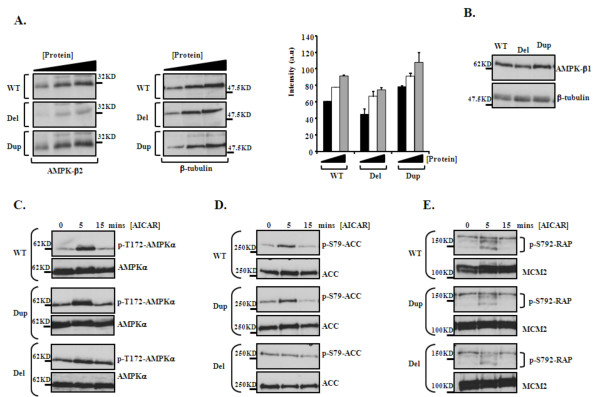
**Functional assays for PRKAB2 in patient cells. (A)**. Left panels:Titrated whole cell extracts wereblotted for AMPKβ2 (encoded by *PRKAB2*) in wild-type (WT),Del and Dup containing LBCs. Right panels:Blots were re-probed with anti-β-tubulin. **Graph: **Densiometric quantification of AMPK-β2 expression from titrated extracts, going from low (black bar), intermediate (white) to higher (grey) amounts of protein, normalized to β-tubulin loading, from three separate determinations (a.u. arbitrary units). p = 0.01 for Del and p < 0.005 for Dup LBCs compared to WT. **(B)**. AMPK subunit AMPK-β1, encoded by the *PRKAB1 *gene on chromosome 12q24.1, shows equivalent expression in the wild-type (WT), Del and Dup containing LBCs. β-tubulin was used to confirm equal loading. **(C)**. AICAR-induced (2 mM) activation of the AMPK kinase was monitored using phosphorylation of the AMPKα subunit on threonine 172 (p-T172-AMPKα). Dup and Del containing LBCs exhibited higher levels of p-T172-AMPKα at the 0 time (untreated), relative to wild-type (WT). Only the 1q21.1 Del containing LBCs appeared to be unresponsive to AICAR-treatment here. Blots were re-probed with for native AMPKα to confirm loading. **(D)**. AICAR-induced (2 mM) activation of AMPK was evaluated by monitoring phosphorylation of the AMPK substrate Acetyl-CoA Carboxylase on serine 79 (p-S79-ACC). Similar to p-T172-AMPKα, the Del containing LBCs appear unresponsive to the AICAR treatment. Blots re-probed for native ACC to confirm loading. **(E)**. AICAR-induced activation of AMPK was also evaluated by phosphorylation of the AMPK substrate RAPTOR on serine 792 (p-S792-RAP) under identical conditions as in (B) and (C). Again, Del containing LBCs appeared somewhat unresponsive to AICAR. Blots re-probed for MCM2 to confirm loading.

Expression of *PRKAB2 *protein product, AMPKβ2, in patient cells was decreased in the cell line with 1q21.1 Del and increased in the cell line with 1q21.1 Dup compared to a wild-type (WT) control, whilst that of the β1 subunit was unaffected (Figure 5A and 5B). The gene encoding AMPK-β1 subunit (*PRKAB1*) is located on chromosome 12q24.1 and so is not within the 1q21.1 CNV. To investigate the impact of increased and decreased AMPK-β2 expression on AMPK activity we treated patient-derived LBCs with AICAR (*N*^1^-(β-D-Ribofuranosyl)-5-aminoimidazole-4-carboxamide), a cell permeable nucleoside analogue that mimics the effects of AMP on the allosteric activation of AMPK, and monitored phosphorylation of AMPK on threonine-172 (p-T172-AMPKα). This is an essential modification, required for and diagnostic of AMPK activity (Figure 5C). Interestingly, both the 1q21.1 Dup and 1q21.1 Del containing LBCs exhibited slightly elevated basal levels of p-T172-AMPKα in the absence of AICAR (0 time), compared to wild-type (WT). Elevated AICAR-induced p-T172-AMPKα was detectable in wild-type LBCs (WT) within 5 minutes, and to a similar extent 1q21.1 Dup containing cells (Figure 5C). In comparison, the change in the AICAR-induced p-T172-AMPKα activity at 5 minutes was less apparent in the 1q21.1 Del containing cell line, and the activity remained constant at 15 minutes. In contrast, the AICAR-induced p-T172-AMPKα activity of the WT and 1q21.1 Dup containing cell line was reduced after 15 minutes. This suggests that decreased AMPK-β2 level is associated with somewhat unresponsive AMPK activation, while the 1q21.1 duplication-containing LBC (Dup) showed similar pattern of responsiveness to WT cells under these conditions (Figure 5C).

To further substantiate these findings we explored AMPK-mediated phosphorylation of two of its well known substrates, Acetyl-CoA Carboxylase (ACC) and RAPTOR. ACC is a key mediator of fatty acid (FA) synthesis. AMPK-induced phosphorylation of ACC on serine-79 (p-S79-ACC) inhibits ACC enzymatic activity thereby limiting FA synthesis under energy limiting conditions (i.e. high [AMP] and low [ATP]) [32]. Consistent with our findings with p-T172-AMPKα, we found efficient induction of p-S79-ACC in WT and LBCs with 1q21.1 Dup within 5 minutes of AICAR treatment, whilst the LBCs with 1q21.1 Del failed to exhibit significant levels of p-S79-ACC under these conditions. This data supports the observation of sub-optimal AMPK activity in this line (Figure 5D).

RAPTOR is an important regulatory component of the mTOR containing complex 1 (mTORC1) and is required for optimal mTOR kinase activity [35]. AMPK-mediated phosphorylation of RAPTOR on serine-792 (p-S792-RAP) inhibits mTORC1 thereby limiting protein synthesis and inducing cell cycle arrest when cellular energy is limiting. Again, consistent with sub-optimal AMPK activity in the 1q21.1 Del containing LBCs, we found reduced AICAR-induced p-S792-RAPTOR in these cells in contrast to the 1q21.1 Dup containing line and the WT control (Figure 5D). Together, these results suggest that haploinsufficiency of *PRKAB2 *results in reduced expression of AMPK-β2 which is associated with impaired AICAR-induced AMPK activation. In contrast, duplication of *PRKAB2 *did not negatively impact on AMPK activity under the conditions examined here.

## Discussion

We have performed whole genome expression and cell function studies in carriers of 1q21.1 deletion and 1q21.1 duplication. Our data show that the top genes ranked based on correlation of expression and 1q21.1 copy number change are significantly enriched for 1q21.1 genes, indicating association of expression and copy number for ~50% of 1q21.1 CNV genes. Furthermore, we show that the function of proteins coded by two of the genes from the 1q21.1 CNV, which ranked highest in 1q21.1 copy number expression correlation, is altered in both the deletion and duplication patient cell lines.

*CHD1L*, the gene which ranked first in the expression/1q21.1 copy number correlation, has been implicated in chromatin remodeling and relaxation as well as DNA damage response [25,26]. Our studies identified a novel role for *CHD1L *in decatenation, which was suspected based on its known chromatin remodeling function, and the defective Topo II decatenation checkpoint demonstrated here in both the 1q21.1 Del and Dup containing patient cell lines.

It is of interest that the DCC defect detected in the 1q21.1 Del and Dup containing cell lines is comparable to that seen in cells from Werner syndrome (OMIM 277700), an autosomal recessive disorder, associated with predisposition to cancer and premature aging, neither of which were noted in our patients. The only overlapping feature, short stature, was noted in 5/6 subjects with the 1q21.1 deletion and also reported in subjects from other cohorts [2]. Previous DCC studies of Werner syndrome and control cells suggested that DCC defect *per se *is not sufficient to cause significant genomic instability, but requires absence or dysfunction of "caretaker" genes such as *ATR, BRCA1 or WRN *[29]. It is possible that in cell lines with 1q21.1 Del and Dup the DCC defect is not accompanied with other deleterious events and thus the threshold for significant spontaneous genomic instability leading to premature cell senescence/cancer predisposition is not met. We have not found evidence of spontaneous chromosome instability in the short term chromosome cultures of our patients nor has this been previously reported for any of the1q21.1 CNV subjects who had routine chromosome analysis. Future studies of the association of *CHD1L *with other genes in decatenation checkpoint mechanism may shed more light on the precise role of *CHD1L *in DCC. So, whilst the phenotypic consequences of defective DCC activation in subjects with a 1q21 CNV are unclear, their cellular phenotype does appear to be consistent with CHD1L dysfunction.

Our findings that the same cellular phenotype is present in both the 1q2.1 Del and Dup containing cell lines, is in keeping with reports [26] that both dosage imbalances of *CHD1L *result in identical cellular effects. Haploinsufficiency and duplication sensitivity is thought to affect genes regulating balanced expression of other genes ("master genes" [36]), which is in keeping with *CHD1L*'s role as a chromatin remodeler and indirect regulator of many key biological processes such as replication, transcription and translation [37]. In that respect, it is interesting to note that > 18 genes with a role in chromatin remodeling have been implicated in intellectual disability [38].

*PRKAB2*, which ranked 10^th ^in the expression/1q21.1 copy number correlation, encodes the β2-subunit of AMPK, a key regulator of cellular response to a large number of external stimuli which modulates energy levels at the cellular and organism level [39]. The deregulation of AMPKβ2 function in 1q21.1 deletion and duplication carriers was suspected based on a) changes in levels of AMPKβ2 protein (in keeping with the 1q21.1 copy number state and expression level of the PRKAB2 gene), b) different basal levels of p-T172-AMPKα in both 1q21.1 Del and Dup containing lines in comparison to WT, and c) sub-optimal AICAR-induced phosphorylation of the AMPK substrates ACC and RAPTOR, which was more obvious in the 1q21.1 Del containing line. The last observation could be explained by the fact that AMPK, as a multi protein complex, may be sensitive to imbalances of its components [36], and that reduced availability of a regulatory β-isoform, as occurs here, could impact on AMPK activity more than over-abundance.

The multifaceted nature of AMPK role in brain function is of particular interest to the 1q21.1 phenotype which most consistently includes some form of learning difficulty. Previous studies showed that alternations of AMPK activity resulted in profound abnormalities of the central nervous system in AMPK-β1^-/- ^knockout mice which had reduction of AMPK activity [34], whereas the consequences of AMPK activation remain controversial as some groups have shown that AMPK activation is neuroprotective while others show that AMPK overactivation is detrimental [39]. The essential role of AMPK in brain function is further supported by its inhibition of the mTOR pathway [32] which is required for learning and memory [40].

Our studies are the first to report that the function of two genes integral to 1q21.1 CNV is changed in patient lymphoblasts and that both genes are likely to be dosage sensitive. Both genes are expressed in multiple tissues, including brain [34,41] which may explain the multi-systemic nature of the physical abnormalities, and the frequent involvement of learning difficulty albeit at a very variable levels. It remains uncertain as to the tissue-specific consequences of gene function changes in individuals with 1q21.1 CNV although AMPK is clearly involved in brain development and homeostasis. We believe that our investigations are unique as they pointed to genes for which further functional investigation in additional carriers and cell lines may prove to be worthwhile.

The phenotypic variability for some CNVs has been traditionally explained by genetic and environmental factors [42]. In that respect it is of interest to note that *CHD1L *and *PRKAB2 *have a role in sensing and responding to genomic (chromosomal structure) and metabolic (energy level) stress and therefore their dysfunction may result in a more severe phenotype in individuals which experienced more adverse environmental conditions during development. Sequence changes of other genes from the 1q21.1 region as well as other genes across the genome that impair their function cannot be ruled out as a source of variability at this time and the new whole genome sequencing technologies will no doubt become useful in future investigations of their contribution to the development of an abnormal phenotype.

## Conclusion

Our studies are unique as they provide evidence of changes in the function of genes from 1q21.1 CNV in lymphoblasts from both deletion and duplication carriers. Furthermore, they also provide evidence that deletions and duplications can have similar (e.g. DCC deficiency in 1q21.1 Del and Dup containing LBCs) as well as differing functional consequences (e.g. less responsive AICAR-induced AMPK activity in 1q21.1 Del containing LBCs) depending on the genes and pathways involved. Our results support the use of patient lymphoblasts for dissecting the functional sequelae of genes integral to CNVs in carrier cell lines, ultimately enhancing understanding of biological processes which may contribute to the clinical phenotype.

## Competing interests

The authors declare that they have no competing interests.

## Authors' contributions

ERS designed the study, initiated the collaborative project, monitored data collection for the whole study, and revised the paper. M.O'D designed the functional analysis for the two genes, and wrote the functional aspect of the paper. ES and CH performed the genomic and expression related experiments and drafted the manuscript. SMartell and YQ contributed to genomic array data analysis. RC and DA performed the functional studies. ME and PP performed the statistical analysis of the expression data. EC, SH, BM, SMarles, AM, AD, SL contributed with clinical information on the subjects. JJA reviewed the manuscript. MH and CT supervised the 2.7 Affymetix genomic analysis. All authors approved and read the final manuscript.

## Supplementary Material

Additional file 1 **Table S1: Clinical and genomic information on subjects included in the study**. **Table S2: **Top 100 Genes from Expression/1q21.1 Copy Number correlation analysis. Click here for file

Additional file 2 **Figure S1: Correlation of expression and 1q21.1 copy number for probes across the genome expressed as log_10 _of p values**. Click here for file
