# Supplementary material for: Understanding the impact of 1q21.1 copy number variant
Source: Orphanet J Rare Dis. 2011 Aug 8;6:54. doi: 10.1186/1750-1172-6-54 (PMC3180300; doi:10.1186/1750-1172-6-54)
Supplement: Additional file 1 — Table S1: Clinical and genomic information on subjects included in the study. Table S2: Top 100 Genes from Expression/1q21.1 Copy Number correlation analysis. [file 1750-1172-6-54-S1.RTF]

Supplementary Table 1: Genomic characteristics of 1q21.1 CNV and their associated phenotypes

Family	Subject	Deletion/    Duplicat-ion	Breakpoints
Original detection method	Breakpoints Affymetrix 2.7M 	FISH clones	Size (kb)	Genes 	Prenatal findings/ newborn period	Mild-Moderate Intellectual Disability (ID)	Language delays	Growth delay	Craniofacial dysmorphisms	Hands and Feet Abnormalities	Heart Anomalies	Eye anomalies	Neurological	Behavioural	Organs/ Systems
defects	
A	1-proband	1q21.1 Deletion	145,110,000 -146,190,000 &	144,966,524 - 146,296,189	RP11-433J22	1,329.67	LOC728989, PRKAB2, PDIA3P, FMO5, CHD1L, BCL9, ACP6, GJA5, GJA8, GPR89B, GPR89C, PDZK1P1, NBPF11	Pregnancy complicated by hyperemesis and pre-eclampsia; mother hospitalized for hypertension, mild edema and thrombocytopenia. Proband born by C section due to maternal hypertension 	Yes	Severe articulation disorder; spoke first words between 14-18 months; put two words together at age 3	Short stature; at age 10 height and weight at 3rd  and head circumference at 10th  percentile	Microcephaly, Doliocephaly, upslanting palpebral fissures, high and prominent forehead, bilateral epicanthal folds, mild retrognathia; long eyelashes, upslanting palpebral fissures	Clinodactyly, prominent fingertip pads, broad thumbs, hypermobile
joints, especially of mid-phalangeal and metatarsal joints	None	None	Headaches associated with photophobia and nausea	Anger  and frustration associated with difficulty to express himself	Grade 4 right kidney reflux with a duplex ureter, tortuous ureter, dilated renal pelvis	
	2-proband's mother	1q21.1 Deletion	FISH 	144,966,524 - 146,296,189	RP11-433J22	1,329.67	LOC728989, PRKAB2, PDIA3P, FMO5, CHD1L, BCL9, ACP6, GJA5, GJA8, GPR89B, GPR89C, PDZK1P1, NBPF11	Born at 37 weeks, and required hospital/
incubator  care for one month	Learning difficulties; repeated grade 2; required
learning assistance until grade 10	No 	Short stature; at 40 years height at 5 percentile, weight at 75 percentile, head circumference at 25th percentile	mild posterior rotation of both ears	Hyperextensibility at mid-phalangeal joints and elbows	Mitral valve anomaly, ventricular septal defect.	None	Memory problems, difficulty expressing thought, migraines	Panic attacks, Restless leg syndrome	Possible Meckel's diver-ticulum removed at 16yrs. Re-current otitis media requiring tympano-stomy tubes, 	
	3-proband's maternal grand-mother	1q21.1 Deletion	FISH 	144,975,442 - 146,297,463	RP11-433J22	1,322.02	LOC728989, PRKAB2, PDIA3P, FMO5, CHD1L, BCL9, ACP6, GJA5, GJA8, GPR89B, GPR89C, PDZK1P1, NBPF11	unknown	No; finished 11/12 grades	No known delay	Short stature; at 59 years 
<3 rd
percentile	None	None	None	None	Migraines	None	None	


B	1-proband	1q21.1 Deletion 	144,798,337-146,290,832**	144,967,161 - 146,296,189	RP11-242B17; RP11-533N14	1,329.03	LOC728989, PRKAB2, PDIA3P, FMO5, CHD1L, BCL9, ACP6, GJA5, GJA8, GPR89B, GPR89C, PDZK1P1, NBPF11	Toxemia, maternal smoking; born at 37 weeks, 3178g; induced VD with forceps after spontaneous ROM; spina bifida occulta L4-5; laminectomy at age 1	Yes; Grade 6 education; PIQ~VIQ at age 26 years	Significant - expressive and receptive; delays  by 5 years; VIQ<PIQ at age 7	Short stature; at 26 years height 3rd and head circumference 50th percentile, weight at 90th percentile	High and prominent forehead, low set simple ears, mild hypertelorism, narrow palpebral fissures, bulbous nose, high arched palate	Small hands, short fingers	None	Strabismus	Schizophrenia	ADHD	Mild hearing loss, recurrent ear infect-ions	
	2-proband's daughter	1q21.1 Deletion 	144,798,337 - 146,290,832**	DNA not available	RP11-242B17	1,492.50	LOC728989, PRKAB2, PDIA3P, FMO5, CHD1L, BCL9, ACP6, GJA5, GJA8, GPR89B, GPR89C, PDZK1P1, NBPF11	Abnormal fetal ultrasound (left polycystic kidney), SVD at 31 weeks, 1790g;  multiple congenital anomalies: L dysplastic kidney, ureterocele, possible separated uterus; RDS with R pneumothorax subepenyman	IQ not formally assessed, but likely in the moderate ID range;
significant fine and gross motor delays; not toilet trained	Significant, first noticed at 18 month; Essentially nonverbal at 7.5 years limited to pointing and few single words only, no sentences. possible imaginary play	Short stature; at age 7.5 years height <3rd percentile and head circumference 40th percentile; 	Complex hair whorl pattern with 5 whorls over occiput, right frontal upsweep to hair, small epicanthal folds	Ectopic hair growth over elbows, broad big toes, deep set nails, fetal pads	None	L amblyopia secondary to esotropia	Hypotonia, mild thinning of corpus callosum on MRI	Possible ASD, focal language delays, outgoing and has good eye contact	Absent left kidney	
	3-proband's brother	1q21.1 Duplicat-ion + 1q21.1 Deletion	144,100,334-144,458,066 (dup)    145,098,652-146,193,043 (del)**	144,102,262 - 144,544,341 (dup) 144,704,815 - 146,296,189 (del)	duplication not confirmed using RP11-315I20; deletion confirmed with RP11-1149B23	442.08 (dup) 1,591.37 (del)	HFE2, TXNIP, POLR3GL, ANKRD34A, LIX1L, RBM8A, GNRHR2, PEX11B, ITGA10, ANKRD35, PIAS3, NUDT17, POLR3C, RNF115, CD160, PDZK1, GPR89A (dup) NBPF11, LOC728989, PRKAB2, PDIA3P, FMO5, CHD1L, BCL9, ACP6, GJA5, GJA8, GPR89B, GPR89C, PDZK1P1, NBPF11 (del)	One of twin boys, the other twin was stillborn; neonatal jaundice treated with phototherapy	Low average IQ when tested in childhood and borderline IQ when tested at age 30; Grade 9 education	Mild delays in speech and language at age 5	At age 30 years, height at 25th, weight at 5th , head <3rd percentile	Microcephaly mild frontal bossing, high palate, bifid uvula, ankyloglossia  	None	None	None	None	ADHD as a child; alcohol and drug abuse as a young adult	None
	
C	1-proband	1q21.1 Duplica-tion	144,510,700 - 146,294,854 *	145,056,290 - 146,297,463	RP11-433J22	1,241.17	PRKAB2, PDIA3P, FMO5, CHD1L, BCL9, ACP6, GJA5, GJA8, GPR89B, GPR89C, PDZK1P1, NBPF11	Normal pregnancy	Yes	First sentences at 3 years of age	At 5 years, height, weight and HC was at the 25th, 50th and 98 percentile; 	Appearance of macrocephaly, ear pit, prominence of the forehead, midfacial hypoplasia, short upturned nose	Persistent toe and  fingertip pads, mild hypotonia	None	None	Normal MRI	Mild ADHD, not treated	Mal-rotated bowel, consti-pation; normal renal ultra-sound	
	2-proband's father	1q21.1 Duplicat-ion	FISH 	144,963,437 - 146,295,976	RP11-433J22	1,332.54	LOC728989, PRKAB2, PDIA3P, FMO5, CHD1L, BCL9, ACP6, GJA5, GJA8, GPR89B, GPR89C, PDZK1P1, NBPF11	unknown	Learning challenges, had to work hard to pass grades, completed grade 12 and had some college training	None	None	None	None	None	None	None	Mild ADHD as a child, not treated	None	

Original array platforms: Nimblegen 385K Whole-Genome Tiling array (§ ), Agilent 105K whole genome oligonucleotide array (*),  Signature Genomics SignatureChipWGTM 1.1 (**); SVD = spontaneous vaginal delivery, PIQ = Performance IQ; VIQ = Verbal IQ RDS = respiratory distress syndrome

Supplementary Table 2: Top 100 Genes from Expression/Copy Number correlation analysis

Gene ID	Gene Name	Chr	Cytoband	Strand	Illumina Probe	Accession	Protein Product	p value	Correlation	
*CHD1L	chromodomain helicase DNA binding protein 1-like (CHD1L), mRNA.	1	1q21.1c	+	ILMN_1786016	NM_004284.3	NP_004275.3	2.42E-05	Positive	
TRIM6	tripartite motif-containing 6 (TRIM6), transcript variant 2, mRNA.	11	11p15.4c	+	ILMN_1656910	NM_058166.3	NP_477514.1	5.53E-05	Positive	
FMNL2	formin-like 2 (FMNL2), mRNA.	2	2q23.3d	+	ILMN_1730491	NM_052905.3	NP_443137.2	7.31E-05	Negative	
C1orf2	chromosome 1 open reading frame 2 (C1orf2), transcript variant 1, mRNA.	1	1q22a	-	ILMN_1795026	NM_006589.2	NP_006580.2	2.02E-04	Positive	
RPL10	ribosomal protein L10 (RPL10), mRNA.	X	Xq28g	+	ILMN_2084182	NM_006013.2	NP_006004.1	2.41E-04	Positive	
HLA-DQA1	PREDICTED: major histocompatibility complex, class II, DQ alpha 1, transcript variant 10 (HLA-DQA1), mRNA.		6p21.32b		ILMN_1808405	XM_936128.2	XP_941221.1	2.73E-04	Positive	
ZNF559	zinc finger protein 559 (ZNF559), mRNA.	19	19p13.2c	+	ILMN_1677785	NM_032497.1	NP_115886.1	2.89E-04	Positive	
EIF1AX	eukaryotic translation initiation factor 1A, X-linked (EIF1AX), mRNA.	X	Xp22.12b	-	ILMN_1813240	NM_001412.3	NP_001403.1	3.44E-04	Negative	
UBD	ubiquitin D (UBD), mRNA.	6	6p22.1a	-	ILMN_1678841	NM_006398.2	NP_006389.1	3.66E-04	Negative	
*PRKAB2	protein kinase, AMP-activated, beta 2 non-catalytic subunit (PRKAB2), mRNA.	1	1q21.1c	-	ILMN_1786021	NM_005399.3	NP_005390.1	5.97E-04	Positive	
SAV1	salvador homolog 1 (Drosophila) (SAV1), mRNA.	14	14q22.1b	-	ILMN_2050654	NM_021818.2	NP_068590.1	6.25E-04	Negative	

SIGLEC10	sialic acid binding Ig-like lectin 10 (SIGLEC10), mRNA.	19	19q13.33d	-	ILMN_1655549	NM_033130.2	NP_149121.2	7.08E-04	Positive	
RSPH1	radial spoke head 1 homolog (Chlamydomonas) (RSPH1), mRNA.	21	21q22.3b	-	ILMN_1684571	NM_080860.2	NP_543136.1	7.08E-04	Positive	
C14orf124	chromosome 14 open reading frame 124 (C14orf124), mRNA.	14	14q12a	-	ILMN_1771629	NM_020195.1	NP_064580.1	7.57E-04	Positive	
PPP2R5A	protein phosphatase 2, regulatory subunit B', alpha isoform (PPP2R5A), mRNA.	1	1q32.3b	+	ILMN_1738784	NM_006243.2	NP_006234.1	8.30E-04	Positive	
LYRM1	LYR motif containing 1 (LYRM1), mRNA.	16	16p12.2c	+	ILMN_1749244	NM_020424.2	NP_065157.1	8.51E-04	Positive	
SLC10A7	solute carrier family 10 (sodium/bile acid cotransporter family), member 7 (SLC10A7), transcript variant 2, mRNA.	4	4q31.22b	-	ILMN_1732489	NM_001029998.2	NP_001025169.1	9.00E-04	Negative	
GPR63	G protein-coupled receptor 63 (GPR63), mRNA.	6	6q16.1f	-	ILMN_1653648	NM_030784.1	NP_110411.1	9.17E-04	Positive	
BCAS1	breast carcinoma amplified sequence 1 (BCAS1), mRNA.	20	20q13.2c	-	ILMN_1733042	NM_003657.1	NP_003648.1	9.42E-04	Positive	
VCAN	versican (VCAN), mRNA.	5	5q14.3a	+	ILMN_1687301	NM_004385.2	NP_004376.2	9.49E-04	Positive	
SH2B2	SH2B adaptor protein 2 (SH2B2), mRNA.	7	7q22.1e	+	ILMN_1669833	NM_020979.2	NP_066189.2	9.86E-04	Negative	
RAP1GDS1	RAP1, GTP-GDP dissociation stimulator 1 (RAP1GDS1), mRNA.	4	4q23a	+	ILMN_2106167	NM_021159.3	NP_066982.2	1.28E-03	Negative	


OAS1	2',5'-oligoadenylate synthetase 1, 40/46kDa (OAS1), transcript variant 1, mRNA. 	12	12q24.13b	+	ILMN_1672606	NM_016816.2	NP_058132.2	1.33E-03	Positive	
BLK	B lymphoid tyrosine kinase (BLK), mRNA.	8	8p23.1b	+	ILMN_1668277	NM_001715.2	NP_001706.2	1.34E-03	Negative	
IDUA	iduronidase, alpha-L- (IDUA), mRNA.	4	4p16.3c	+	ILMN_1703041	NM_000203.3	NP_000194.2	1.35E-03	Negative	
GMEB2	glucocorticoid modulatory element binding protein 2 (GMEB2), mRNA.	20	20q13.33e	-	ILMN_1683204	NM_012384.2	NP_036516.1	1.38E-03	Positive	
BET1	blocked early in transport 1 homolog (S. cerevisiae) (BET1), mRNA.	7	7q21.3a	-	ILMN_1684042	NM_005868.4	NP_005859.1	1.43E-03	Negative	
DSCR6	Down syndrome critical region gene 6 (DSCR6), mRNA.	21	21q22.13a	+	ILMN_1709257	NM_018962.1	NP_061835.1	1.43E-03	Positive	
DLG3	discs, large homolog 3 (neuroendocrine-dlg, Drosophila) (DLG3), transcript variant 1, mRNA.	X	Xq13.1c	+	ILMN_2336728	NM_021120.2	NP_066943.2	1.47E-03	Positive	
*GPR89A	G protein-coupled receptor 89A (GPR89A), mRNA.	1	1q21.1d	+	ILMN_2116594	NM_016334.2	NP_057418.1	1.55E-03	Positive	
RPL23AP13	ribosomal protein L23a pseudogene 13 (RPL23AP13), non-coding RNA.	2	2p16.2a	+	ILMN_2124757	NR_002229.1		1.64E-03	Negative	
MRFAP1	Mof4 family associated protein 1 (MRFAP1), mRNA.	4	4p16.1f	+	ILMN_2055165	NM_033296.1	NP_150638.1	1.74E-03	Positive	
BTBD12	BTB (POZ) domain containing 12 (BTBD12), mRNA.	16	16p13.3c	-	ILMN_1732885	NM_032444.2	NP_115820.2	1.74E-03	Positive	


TUBB4Q	tubulin, beta polypeptide 4, member Q (TUBB4Q), mRNA.	4	4q35.2d	-	ILMN_1750100	NM_020040. 3	NP_064424.3	1.75E-03	Positive	
ZNF626	zinc finger protein 626 (ZNF626), transcript variant 2, mRNA.	19	19p12d	-	ILMN_1794823	NM_145297.3	NP_660340.1	1.81E-03	Positive	
COL24A1	collagen, type XXIV, alpha 1 (COL24A1), mRNA.	1	1p22.3d	-	ILMN_1810996	NM_152890.4	NP_690850.1	1.85E-03	Positive	
ACAP3	ArfGAP with coiled-coil, ankyrin repeat and PH domains 3 (ACAP3), mRNA.	1	1p36.33a	-	ILMN_1743847	NM_030649.1	NP_085152.1	1.88E-03	Negative	
EFHA1	EF-hand domain family, member A1 (EFHA1), mRNA.	13	13q12.11c	-	ILMN_1738346	NM_152726.1	NP_689939.1	1.91E-03	Negative	
SH3BGRL3	SH3 domain binding glutamic acid-rich protein like 3 (SH3BGRL3), mRNA.	1	1p36.11b	+	ILMN_1737163	NM_031286.3	NP_112576.1	1.92E-03	Negative	
FYN	FYN oncogene related to SRC, FGR, YES (FYN), transcript variant 2, mRNA.	6	6q21i	-	ILMN_1781207	NM_153047.1	NP_694592.1	1.98E-03	Negative	
LAMP2	lysosomal-associated membrane protein 2 (LAMP2), transcript variant LAMP2A, mRNA.	X	Xq24d	-	ILMN_2243687	NM_002294.1	NP_002285.1	2.00E-03	Negative	
*BCL9	B-cell CLL/lymphoma 9 (BCL9), mRNA.	1	1q21.1c	+	ILMN_1704452	NM_004326.2	NP_004317.2	2.01E-03	Positive	
CPXM1	carboxypeptidase X (M14 family), member 1 (CPXM1), mRNA.	20	20p13c	-	ILMN_1712046	NM_019609.3	NP_062555.1	2.13E-03	Positive	
TUBB6	tubulin, beta 6 (TUBB6), mRNA.	18	18p11.21e	+	ILMN_1702636	NM_032525.1	NP_115914.1	2.21E-03	Positive	


C4BPB	complement component 4 binding protein, beta (C4BPB), transcript variant 3, mRNA.	1	1q32.2a	+	ILMN_1694588	NM_001017365.1	NP_001017365.1	2.27E-03	Positive	
COMMD1	copper metabolism (Murr1) domain containing 1 (COMMD1), mRNA.	2	2p15c	+	ILMN_1761242	NM_152516.2	NP_689729.1	2.41E-03	Positive	
KIAA0247	KIAA0247 (KIAA0247), mRNA.	14	14q24.1e	+	ILMN_2226917	NM_014734.2	NP_055549.1	2.65E-03	Negative	
NUBP1	nucleotide binding protein 1 (MinD homolog, E. coli) (NUBP1), mRNA.	16	16p13.13d	+	ILMN_1689342	NM_002484.2	NP_002475.2	2.69E-03	Positive	
SMG7	Smg-7 homolog, nonsense mediated mRNA decay factor (C. elegans) (SMG7), transcript variant 4, mRNA.	1	1q25.3e	+	ILMN_2368597	NM_201569.1	NP_963863.1	2.98E-03	Negative	
CRYZ	crystallin, zeta (quinone reductase) (CRYZ), mRNA.	1	1p31.1h	-	ILMN_1672389	NM_001889.2	NP_001880.2	3.07E-03	Positive	
VAMP5	vesicle-associated membrane protein 5 (myobrevin) (VAMP5), mRNA.	2	2p11.2f	+	ILMN_1809467	NM_006634.2	NP_006625.1	3.17E-03	Negative	
MMP7	matrix metallopeptidase 7 (matrilysin, uterine) (MMP7), mRNA.	11	11q22.2a	-	ILMN_2192072	NM_002423.3	NP_002414.1	3.22E-03	Positive	
ACAD10	acyl-Coenzyme A dehydrogenase family, member 10 (ACAD10), mRNA.	12	12q24.12b	+	ILMN_1687303	NM_025247.4	NP_079523.3	3.23E-03	Positive	


LDHC	lactate dehydrogenase C (LDHC), transcript variant 2, mRNA.	11	11p15.1c	+	ILMN_2326324	NM_017448.1	NP_059144.1	3.29E-03	Negative	


PIP5K1C	phosphatidylinositol-4-phosphate 5-kinase, type I, gamma (PIP5K1C), mRNA.	19	19p13.3e	-	ILMN_1668514	NM_012398.1	NP_036530.1	3.29E-03	Negative	
XRN2	5'-3' exoribonuclease 2 (XRN2), mRNA.	20	20p11.22b	+	ILMN_2196479	NM_012255.3	NP_036387.2	3.30E-03	Negative	
JMJD1B	jumonji domain containing 1B (JMJD1B), mRNA.	5	5q31.2c	+	ILMN_1706539	NM_016604.3	NP_057688.2	3.32E-03	Positive	
KIF3B	kinesin family member 3B (KIF3B), mRNA.	20	20q11.21b	+	ILMN_2081398	NM_004798.2	NP_004789.1	3.44E-03	Negative	
RASSF6	Ras association (RalGDS/AF-6) domain family member 6 (RASSF6), transcript variant 1, mRNA.	4	4q13.3d	-	ILMN_1657381	NM_177532.3	NP_803876.1	3.45E-03	Positive	
*PDIA3P	protein disulfide isomerase family A, member 3 pseudogene (PDIA3P), non-coding RNA.	1	1q21.1c	+	ILMN_2075436	NR_002305.1		3.48E-03	Negative	
UCP2	uncoupling protein 2 (mitochondrial, proton carrier) (UCP2), nuclear gene encoding mitochondrial protein, mRNA.	11	11q13.4b	-	ILMN_1685625	NM_003355.2	NP_003346.2	3.52E-03	Negative	
ITM2A	integral membrane protein 2A (ITM2A), mRNA.	X	Xq21.1b	-	ILMN_2076602	NM_004867.3	NP_004858.1	3.75E-03	Negative	
GPR56	G protein-coupled receptor 56 (GPR56), transcript variant 1, mRNA.	16	16q13d	+	ILMN_1697228	NM_005682.4	NP_005673.3	3.80E-03	Negative	


ILDR1	immunoglobulin-like domain containing receptor 1 (ILDR1), mRNA.	3	3q13.33c	-	ILMN_2043079	NM_175924.2	NP_787120.1	3.91E-03	Negative	
SUMF2	sulfatase modifying factor 2 (SUMF2), transcript variant 4, mRNA.	7	7p11.2b	+	ILMN_1685371	NM_001042470.1	NP_001035935.2	3.95E-03	Negative	
FLJ22662	hypothetical protein FLJ22662 (FLJ22662), mRNA.	12	12p13.1a	-	ILMN_1707286	NM_024829.4	NP_079105.3	3.99E-03	Positive	
EIF2C1	eukaryotic translation initiation factor 2C, 1 (EIF2C1), mRNA.	1	1p34.3e-p34.3d	+	ILMN_1671326	NM_012199.2	NP_036331.1	4.20E-03	Positive	

COL4A4	collagen, type IV, alpha 4 (COL4A4), mRNA.	2	2q36.3b	-	ILMN_1778308	NM_000092.4	NP_000083.3	4.25E-03	Negative	
LRRC8B	leucine rich repeat containing 8 family, member B (LRRC8B), mRNA.	1	1p22.2c	+	ILMN_1712128	NM_015350.1	NP_056165.1	4.35E-03	Negative	
TRAPPC6A	trafficking protein particle complex 6A (TRAPPC6A), mRNA.	19	19q13.32a	-	ILMN_1775703	NM_024108.1	NP_077013.1	4.38E-03	Positive	
B3GNT4	UDP-GlcNAc:betaGal beta-1,3-N-acetylglucosaminyltransferase 4 (B3GNT4), mRNA.	12	12q24.31c	+	ILMN_1771260	NM_030765.2	NP_110392.1	4.40E-03	Negative	
TCL1B	T-cell leukemia/lymphoma 1B (TCL1B), transcript variant 1, mRNA.	14	14q32.13b	+	ILMN_2382309	NM_004918.2	NP_004909.1	4.51E-03	Positive	
GIMAP2	GTPase, IMAP family member 2 (GIMAP2), mRNA.	7	7q36.1c	+	ILMN_2135272	NM_015660.2	NP_056475.1	4.58E-03	Negative	
CTTN	cortactin (CTTN), transcript variant 2, mRNA.	11	11q13.3c-q13.3d	+	ILMN_2393712	NM_138565.1	NP_612632.1	4.66E-03	Positive	


TARS	threonyl-tRNA synthetase (TARS), mRNA.	5	5p13.3a	+	ILMN_1685480	NM_152295.3	NP_689508.3	4.72E-03	Negative	
MYH10	myosin, heavy chain 10, non-muscle (MYH10), mRNA.	17	17p13.1c	-	ILMN_1815154	NM_005964.1	NP_005955.1	4.77E-03	Positive	
FAM20C	family with sequence similarity 20, member C (FAM20C), mRNA.		7p22.3d		ILMN_1712684	NM_020223.2	NP_064608.2	4.87E-03	Negative	
OMG	oligodendrocyte myelin glycoprotein (OMG), mRNA.	17	17q11.2d	-	ILMN_1739235	NM_002544.3	NP_002535.3	4.88E-03	Positive	
MYO1D	myosin ID (MYO1D), mRNA.	17	17q11.2e	-	ILMN_1805999	NM_015194.1	NP_056009.1	5.12E-03	Negative	
H2AFY	H2A histone family, member Y (H2AFY), transcript variant 2, mRNA.	5	5q31.1f	-	ILMN_2275437	NM_004893.2	NP_004884.1	5.12E-03	Negative	
OSBPL2	oxysterol binding protein-like 2 (OSBPL2), transcript variant 2, mRNA.	20	20q13.33c	+	ILMN_1656482	NM_144498.1	NP_653081.1	5.13E-03	Negative	
*ACP6	acid phosphatase 6, lysophosphatidic (ACP6), mRNA.	1	1q21.1c	-	ILMN_2234343	NM_016361.2	NP_057445.2	5.14E-03	Positive	
DCTN6	dynactin 6 (DCTN6), mRNA.	8	8p12e	+	ILMN_2204983	NM_006571.2	NP_006562.1	5.23E-03	Negative	
BIRC3	baculoviral IAP repeat-containing 3 (BIRC3), transcript variant 1, mRNA.	11	11q22.2a	+	ILMN_1776181	NM_001165.3	NP_001156.1	5.31E-03	Positive	
IQCB1	IQ motif containing B1 (IQCB1), transcript variant 3, mRNA.	3	3q13.33c	-	ILMN_2316104	NM_001023571.1	NP_001018865.1	5.44E-03	Negative	
TBRG4	transforming growth factor beta regulator 4 (TBRG4), transcript variant 2, mRNA.	7	7p13c	-	ILMN_2414848	NM_030900.2	NP_112162.1	5.53E-03	Positive	


RTKN	rhotekin (RTKN), transcript variant 2, mRNA.	2	2p13.1b	-	ILMN_1680591	NM_033046.2	NP_149035.1	5.54E-03	Positive	
ATMIN	ATM interactor (ATMIN), mRNA.	16	16q23.2b	+	ILMN_2223720	NM_015251.2	NP_056066.2	5.66E-03	Negative	
UBE2D4	ubiquitin-conjugating enzyme E2D 4 (putative) (UBE2D4), mRNA.	7	7p13e	+	ILMN_1707084	NM_015983.2	NP_057067.1	5.85E-03	Positive	
TBX2	T-box 2 (TBX2), mRNA.	17	17q23.2b	+	ILMN_1792256	NM_005994.3	NP_005985.3	5.92E-03	Negative	
ACSM3	acyl-CoA synthetase medium-chain family member 3 (ACSM3), transcript variant 1, mRNA.	16	16p12.2c	+	ILMN_1685952	NM_005622.3	NP_005613.2	5.94E-03	Positive	
ITGB1	integrin, beta 1 (fibronectin receptor, beta polypeptide, antigen CD29 includes MDF2, MSK12) (ITGB1), transcript variant 1A, mRNA.	10	10p11.22b	-	ILMN_1723467	NM_002211.2	NP_002202.2	6.02E-03	Negative	
FCRLB	Fc receptor-like B (FCRLB), mRNA.	1	1q23.3b	+	ILMN_1782015	NM_001002901.2	NP_001002901.1	6.20E-03	Negative	
TMEM30A	transmembrane protein 30A (TMEM30A), mRNA.	6	6q14.1a	-	ILMN_1735680	NM_018247.2	NP_060717.1	6.49E-03	Positive	
DPP4	dipeptidyl-peptidase 4 (CD26, adenosine deaminase complexing protein 2) (DPP4), mRNA.	2	2q24.2d	-	ILMN_1692535	NM_001935.3	NP_001926.2	6.56E-03	Negative	
PBLD	phenazine biosynthesis-like protein domain containing (PBLD), transcript variant 2, mRNA.	10	10q21.3d	-	ILMN_2304404	NM_001033083.1	NP_001028255.1	6.81E-03	Positive	


COX10	COX10 homolog, cytochrome c oxidase assembly protein, heme A: farnesyltransferase (yeast) (COX10), nuclear gene encoding mitochondrial protein, mRNA.	17	17p12b	+	ILMN_1670901	NM_001303.2	NP_001294.2	6.82E-03	Positive	
CAMK1	calcium/calmodulin-dependent protein kinase I (CAMK1), mRNA.	3	3p25.3c	-	ILMN_2140990	NM_003656.3	NP_003647.1	6.88E-03	Negative	
PGCP	plasma glutamate carboxypeptidase (PGCP), mRNA.	8	8q22.1d-q22.1e	+	ILMN_2058795	NM_016134.2	NP_057218.1	6.91E-03	Negative	
ERC1	ELKS/RAB6-interacting/CAST family member 1 (ERC1), transcript variant beta, mRNA.	12	12p13.33c	+	ILMN_2342068	NM_178037.1	NP_829881.1	6.92E-03	Negative	

Genes from the minimal 1q21.1 CNV region are indicated with an * before the gene symbol and are in bold font.
