# Supplementary figures and images for: Understanding the impact of 1q21.1 copy number variant
Source: Orphanet J Rare Dis. 2011 Aug 8;6:54. doi: 10.1186/1750-1172-6-54 (PMC3180300; doi:10.1186/1750-1172-6-54)

## Slide 1
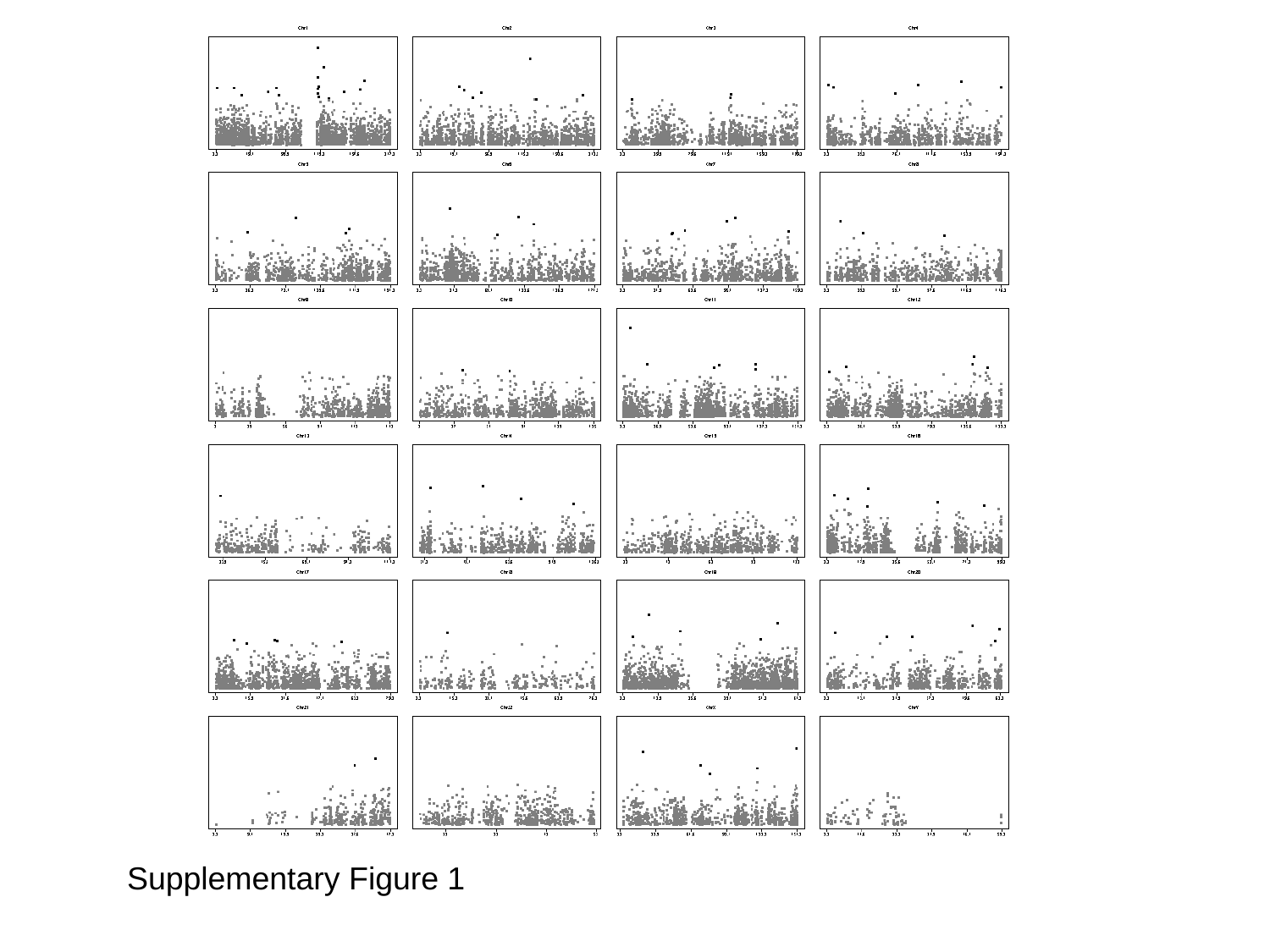

Supplementary Figure 1

Supplement: Additional file 2 — Figure S1: Correlation of expression and 1q21.1 copy number for probes across the genome expressed as log10 of p values. [file 1750-1172-6-54-S2.PPT]
